# Neuroplasticity in autism spectrum disorder: a systematic review

**DOI:** 10.1590/1980-5764-DN-2024-0182

**Published:** 2025-06-02

**Authors:** Gabriela Garcia de Carvalho Laguna, Ana Beatriz Ferreira Gusmão, Breno Oliveira Marques, Níkolas Brayan da Silva Bragas, Isadora Bagues Rodrigues, Rafaela Souza Melo, Katiene Rodrigues Menezes de Azevedo

**Affiliations:** 1 Universidade Federal da Bahia, Instituto Multidisciplinar em Saúde, Vitória da Conquista BA, Brazil.; 2 Universidade Estadual de Santa Cruz, Vitória da Conquista BA, Brazil.

**Keywords:** Autism Spectrum Disorder, Neuronal Plasticity, Neurobiology, Psychiatry, Neurodevelopmental Disorders, Transtorno do Espectro Autista, Plasticidade Neuronal, Neurobiologia, Psiquiatria, Transtornos do Neurodesenvolvimento

## Abstract

The brain's ability to adapt in response to stimuli is called neuroplasticity.

**Objective:**

This study investigates neuroplasticity in autistic individuals, focusing on neurobiological aspects, clinical correlations, and therapeutic interventions.

**Methods:**

This systematic review, registered in the International Prospective Register of Systematic Reviews—PROSPERO (ID: CRD42024522425) and guided by Preferred Reporting Items for Systematic Reviews and Meta-Analyses—PRISMA (2020) criteria, searched databases like Web of Science, Scopus, United States National Library of Medicine/ Medical Literature Analysis and Retrieval System Online (PubMed/Medline), Latin American and Caribbean Health Sciences Literature (LILACS), and Scientific Electronic Library Online (SciELO) for original articles published in 2018–2023.

**Results:**

Of the 2,316 studies found, 11 were selected, involving 1,943 autistic individuals, both children and adults. Most studies were classified as high/moderate quality using the Newcastle-Ottawa and Jadad scales. Observations included variations in SHANK2 gene expression, lower concentrations of α-synuclein, higher β-synuclein in children with autism spectrum disorder (ASD), correlations between NCAM1 expression and motor skills, and higher brain-derived neurotrophic factor (BDNF) concentration compared to non-autistic children.

**Conclusions:**

Alterations in SHANK2, α-synuclein, β-synuclein, NCAM1, and BDNF in ASD suggest biomarkers and therapeutic targets for more effective interventions, improving care for autistic individuals.

## RESUMO.

A capacidade do cérebro adaptar-se em resposta a estímulos é denominada neuroplasticidade.

**Objetivo:**

Este estudo investiga a neuroplasticidade em indivíduos autistas, focando em aspectos neurobiológicos, correlações clínicas e intervenções terapêuticas.

**Métodos:**

Esta revisão sistemática, registrada no Prospective Register of Systematic Reviews — PROSPERO (ID: CRD42024522425) e orientada pelos critérios do Preferred Reporting Items for Systematic Reviews and Meta-Analyses — PRISMA (2020), pesquisou em bases de dados como Web of Science, Scopus, Biblioteca Nacional de Medicina dos Estados Unidos/ Sistema Online de Busca e Análise de Literatura Médica (PubMed/Medline), Literatura Latino-Americana e do Caribe em Ciências da Saúde (LILACS) e Scientific Electronic Library Online (SciELO) por artigos originais publicados em 2018–2023.

**Resultados:**

Dos 2.316 estudos encontrados, 11 foram selecionados, envolvendo 1.943 indivíduos autistas, tanto crianças quanto adultos. A maioria dos estudos foi classificada como de alta/moderada qualidade usando as escalas de Newcastle-Ottawa e Jadad. Observações incluíram variações na expressão do gene SHANK2, menores concentrações de α-sinucleína, maiores de β-sinucleína em crianças com transtorno do espectro autista (TEA), correlações entre a expressão de NCAM1 e habilidades motoras, e maior concentração de fator neurotrófico derivado do cérebro (BDNF) em comparação a crianças não autistas.

**Conclusões:**

Alterações em SHANK2, α-sinucleína, β-sinucleína, NCAM1 e BDNF no TEA sugerem biomarcadores e alvos terapêuticos para intervenções mais eficazes, aprimorando o cuidado de indivíduos autistas.

## INTRODUCTION

Autism spectrum disorder (ASD) is defined as a neurodevelopmental disorder by the Diagnostic and Statistical Manual of Mental Disorders (DSM-5)^1^ and the International Statistical Classification of Diseases and Related Health Problems (ICD-11)^2^. ASD is characterized by impairments in social communication and by restricted and repetitive patterns of behavior, interests, or activities, in varying degrees, encompassing both individuals with intellectual disabilities and/or more limited linguistic abilities, as well as those with above-average intelligence and language skills, though they still maintain some difficulty in communication and social interaction^3,4^.

The diversity and complexity of ASD presentations relate to its complex pathophysiology, influenced by genetic mutations, environmental factors, and exposures from before birth, and it is associated with the availability of resources and services that allow for greater or lesser stimulation of the child^4,5^. The estimated prevalence of ASD is higher in males than in females. However, recent studies indicate a possible lower sensitivity of instruments in identifying autism in girls compared to boys. Additionally, autistic women possibly experience a greater "camouflage" effect than men, due to a neural self-representation response. This effect is related to a brain-behavior response in the social brain function in autism, which is greater in women, leading them to have a higher propensity to hide social symptoms that would facilitate diagnosis, thus underestimating this prevalence^6–9^.

Neuroplasticity relates to the brain's ability to adapt and change in response to stimuli and is a process that demands structural and functional transformations. It involves mechanisms like neuronal regeneration, which relates to synaptic and neurogenic plasticity, and functional regeneration, which includes processes like equipotentiality, vicariation, and diaschisis. Thus, the modifications comprising neuroplasticity are not limited to morphological changes but also functional ones, with biochemical, pharmacological, and neural network alterations, among others^10,11^. In this context, synaptic plasticity is fundamental for learning, memory, and development, as it regulates "neuronal and network activity," and controls synaptic strengths in response to excitation or inhibition, leading to adaptive behaviors and modifying the transfer and processing of information within neural circuits^12–14^. Hebbian plasticity strengthens synaptic connections when there is temporal coincidence between pre- and post-synaptic activity, making it fundamental for encoding information and responses to specific stimuli. However, unregulated Hebbian mechanisms can lead to synaptic saturation and destabilization of neural networks^15,16^.

To counterbalance this, homeostatic plasticity acts as a compensatory mechanism, maintaining neural network stability by adjusting synaptic strength and excitability to prevent runaway synaptic activity. This interplay between Hebbian and homeostatic mechanisms ensures both learning and the preservation of network stability, a balance that is particularly relevant in the context of ASD, where atypical neural connectivity and adaptation are core features^17,18^.

Given this, other reviews have been conducted, contributing to scientific knowledge about the relationship between neuroplasticity and ASD. These studies highlight the aspect of neuroinflammation and its repercussions on ASD, negative adaptation mechanisms, genetic mutations, specific biological components, and other associated pathologies, mainly developed with animal models^19–25^. However, a gap in the literature regarding neurobiological aspects associated with neuroplasticity in ASD is evident, in a more comprehensive manner, beyond specific markers and centered on humans; thus, this review aims to analyze the structural dimension of neuroplasticity in ASD, drawing on a theoretical framework that integrates neurobiological mechanisms with clinical and therapeutic considerations. By emphasizing structural neuroplasticity, we seek to bridge the existing gap and contribute to a more comprehensive understanding of ASD.

## METHODS

This is a systematic review, registered on the International Prospective Register of Systematic Reviews (PROSPERO)^26^ platform, ID: CRD42024522425, aiming to ensure evidence, avoid duplication, improve methods, and reduce biases in health decisions^27^; and guided by the investigative question: "What are the main pieces of evidence (I) regarding neuroplasticity (Co) in autistic individuals (P)?", according to the "Population, Interest/phenomenon of interest, and Context" (PICo) strategy^28^. For its development, the criteria of the Preferred Reporting Items for Systematic Reviews and Meta-Analyses (PRISMA, 2020) were followed^28,29^.

Original studies published between 2018 and 2023 were included, and duplicate studies, non-article text genres, incomplete articles, animal studies, case reports, articles that did not meet the inclusion criteria and/or did not answer the research question for not describing aspects related to neuroplasticity in individuals with ASD, or for being literature reviews, were excluded. Language was not an exclusion criterion, and articles not fully available were accessed through the institution of the authors of this research when available; otherwise, they were requested from the authors.

Searches were conducted in the databases Web of Science, Scopus, United States National Library of Medicine/ Medical Literature Analysis and Retrieval System Online (PubMed/Medline), Latin American and Caribbean Health Sciences Literature (LILACS), and Scientific Electronic Library Online (SciELO) in March 2024. For this, the Health Sciences Descriptors were combined in two search strategies with the Boolean operator AND: i) (Neuronal Plasticity) AND (Autism Spectrum Disorder) AND (Neurobiology); ii) (Neuronal Plasticity) AND (Autism Spectrum Disorder).

The free web platform Rayyan^30^ was used for screening the studies. Screening was conducted in two stages: i) reading of titles and abstracts; ii) reading of eligible full texts; by two independent and anonymized reviewers (GGCL and ABFG), and discrepancies were resolved by consensus. Microsoft Excel was used for data management, extraction, and analysis, including: author and year of publication, study design and location, sample, and main results.

The quality analysis of observational studies was performed using the Newcastle-Ottawa Scale (NOS)^31^ criteria, and that of clinical trials using the Jadad scale^32^. Studies were standardized to be classified as follows: "high quality" (<5/10 points), "intermediate quality" (5–6/10 points), or "low quality" (>6/10 points).

Quantitative information from the research was presented using descriptive statistics, in absolute numbers and percentages, and qualitative information through an individual summary table of the studies and a summary figure of the results of this review.

## RESULTS

**Figure 1:**
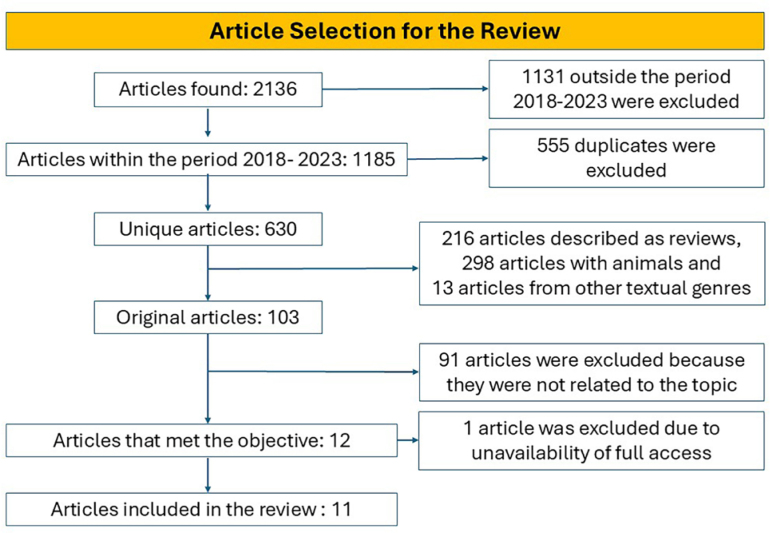
Screening flowchart of the studies.

A total of 2,316 studies were found, of which 11 were selected to compose the bibliographic sample for this research according to the eligibility criteria. Figure 1 illustrates the screening process. Among the included studies, the following countries were represented: United States (18.18%), Canada (18.18%), China (18.18%), Thailand (9.09%), Qatar and Spain (9.09%), Japan (9.09%), Turkey (9.09%), and the United Kingdom (9.09%).

The population sample covered approximately 1,943 individuals (adults and children), with four studies (36.36%) focusing on children, three (27.27%) focusing on mixed populations, and four (36.36%) on adults. The diagnosis of ASD, conducted using the Diagnostic and Statistical Manual of Mental Disorders IV/V—DSM-IV/V criteria, the Autism Diagnostic Interview-Revised (ADI-R), and the Autism Diagnostic Observation Schedule (ADOS) by qualified professionals, was described in ten (90.91%) studies.

Aspects related to neuroplasticity in ASD were addressed, with five studies (45.45%) based on blood plasma evaluation, two (18.18%) on genetic evaluation, and four (36.36%) on other complementary examinations such as magnetic resonance imaging, functional magnetic resonance imaging, electroencephalogram, and repetitive transcranial magnetic stimulation. Supplementary Material 1 (available at https://www.demneuropsy.com.br/wp-content/uploads/2025/02/DN-2024.0182-Supplementary-Material.docx) displays the individual characterization of the studies.

In detail, the approach and investigation of neuroplasticity in patients with ASD in the sample of studies analyzed in this research were highly heterogeneous. This methodological variability may hinder the generalization of results, making it necessary to interpret the findings with caution. Some studies investigated the relationship of the concentration of specific plasma proteins^33–37^. The proteins studied included α-synuclein, β-synuclein, NCAM, brain-derived neurotrophic factor (BDNF), N, N-dimethylglycine, and other advanced glycation end-products (AGEs). Other studies focused on investigating the influence of genetic factors and their correlation with neural plasticity in these patients^38,39^. Twenty-seven miRNAs of patients with ASD were studied for their relationship with the interleukins IL-1ß/IL-10; another genetic aspect studied was the presence of mutations in the SHANK2 gene and its relation to ASD. Additional research produced data from neurosensory tests in patients with ASD^40–42^. The tests involved high-frequency stimulation (HFS) evaluated by persistent changes in visual evoked potentials (VEPs), repetitive transcranial magnetic stimulation (rTMS), and assessment through functional magnetic resonance imaging of the status of visual and auditory repetition suppression, as well as the response to faces, objects, printed words, and spoken words. One study assessed possible neuroanatomical alterations in patients with ASD based on imaging exams^43^.

### Neurobiological aspects

Regarding the studied proteins, α-synuclein levels were significantly lower (p<0.001) in children with ASD compared to the control group, suggesting a possible role of this protein in the pathophysiology of ASD. Conversely, β-synuclein levels were significantly higher (p<0.05), although the clinical relevance of these levels remains poorly defined. Additionally, NCAM1 exhibited a positive, albeit weak, correlation with gross motor skills (p=0.41) and the developmental quotient (p=0.030) in children with ASD^33^. In parallel, NCAM1 was positively correlated, albeit weak, with gross motor skills (p=0.41) and the development quotient (p=0.030) in children with ASD^34^. Another study evaluated the levels of BDNF in patients with ASD and reported that the levels of this protein were significantly higher in the study group compared to the control group (p<0.001)^35^. Additionally, the investigation of advanced glycation end-products (AGEs)—Nε-carboxymethyl-lysine (CML), Nω-carboxymethylarginine (CMA), and hydroimidazolone derived from 3-deoxyglucosone (3DG-H), and the oxidative damage marker o,o’-dityrosine (DT)—in these patients indicates a possible relationship between ASD, increased lipid peroxidation, neuronal plasticity, and proteotoxic stress^37^.

From a genetic perspective, it was observed that the genes targeted by miRNAs are enriched in specific signaling pathways such as neuronal development and synaptic plasticity in all ASD subgroups analyzed in the study^38^. It was also described that, in patients with ASD, reduced dosage or mutation of the SHANK2 gene causes an increase in synaptic connectivity, with a consequent increase in dendrite length and complexity, the number of synapses, and the frequency of spontaneous excitatory postsynaptic currents^39^.

Regarding anatomical changes, a cross-sectional study analyzing cranial magnetic resonance images described that patients with ASD exhibited a 7-year delay in reaching the maximum value compared to the control group in the transverse trajectories of network characteristics. They exhibited inverted U-shaped forms and significant differences in network characteristics at the age of 18 in most densities^43^.

### Clinical correlations

The results were related to the clinical profile of individuals with ASD in 27.27% of the studies^34,40,42^. Through an analysis of the effect of high-frequency visual stimulation on changes in plasticity, measured by changes in visual evoked potentials (VEPs), a reduction in short-term potentiation of VEPs was observed in individuals with ASD. These findings indicate that cortical visual plasticity in ASD may be atypical, with a possible deficiency in its maintenance, characterizing cortical hypoplasticity. Furthermore, a significant association (p=0.044) was found between visual hypersensitivity and the degree of potentiation^40^.

Regarding neuropsychological and motor development, one study associated the levels of a neuronal cell adhesion molecule, NCAM1, in the plasma, reporting a significant reduction in individuals with ASD (p=0.035). There was also a positive correlation between the gross motor skills of children with ASD (p=0.41) and the development quotient (p=0.03)^34^.

In the field of sociability, an evaluation involving faces, objects, and words noted a reduction in face repetition suppression in autistic individuals (p=0.04), with lower repetition suppression being associated with higher severity scores (p<0.05). These changes were related to greater challenges in communication and social interaction; however, such associations are preliminary and require validation in larger samples^42^.

### Intervention possibilities related to neuroplasticity

**Figure 2:**
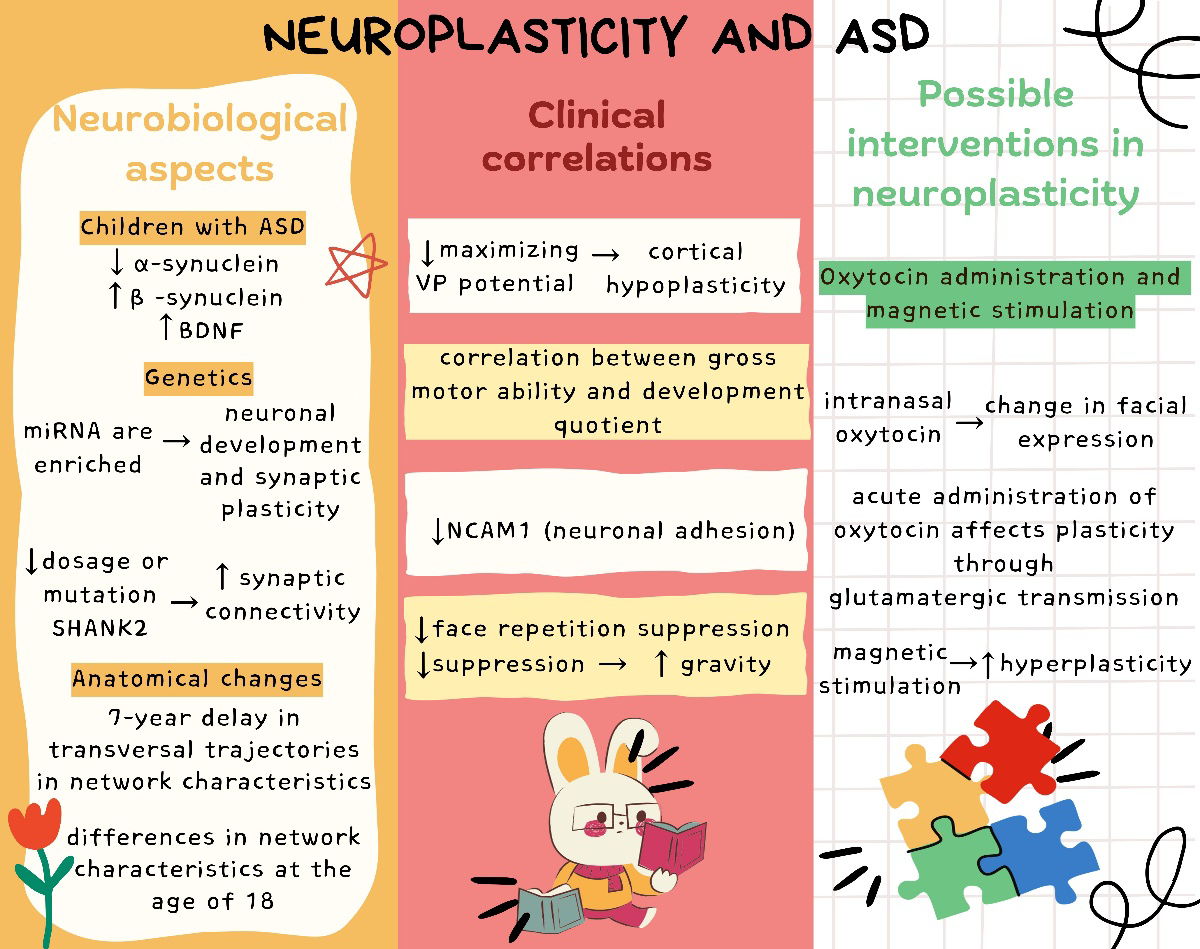
Summary of results.

Some studies proposed measures such as oxytocin administration and rTMS that could influence neuroplasticity to some extent. However, it is worth noting that these approaches remain experimental, and further studies are required to confirm their clinical benefits^36,41^. Intranasal administration of oxytocin was significantly associated with an increase in N,N-dimethylglycine (p=0.043), correlated with changes in the facial expression characteristics of individuals with ASD (p=0.026), indicating an improvement in the pattern of such expressions. Additionally, the study suggested an association between acute oxytocin administration and N-Methyl-D-Aspartate Receptors (NMDAR), impacting neuroplasticity through glutamatergic transmission^36^. Regarding rTMS therapy, hyperplasticity was observed in individuals with ASD due to increased long-term depression (p=0.004) and long-term potentiation (p=0.024). The intervention allowed for a significant attenuation of long-term depression in individuals with ASD (p=0.025)^41^. Figure 2 summarizes the results.

### Quality analysis

Tables 1 and 2 describe the individual quality analysis of the studies. Among the reviewed studies, approximately 54.54% were classified as having high methodological quality, about 27.27% as having moderate methodological quality, and 18.18% as having low methodological quality.

**Table 1:** Quality analysis based on the Newcastle-Ottawa Scale tool.

Studies	Items								
	Selection				Comparability	Exposure			Score
	1	2	3	4	1	1	2	3	
^28^	*	*	-	*	**	-	*	*	7/9
^29^	*	-	-	*	**	–	*	*	6/9
^30^	*	*	*	*	**	*	*	-	8/9
^32^	*	*	*	*	*	*	*	*	8/9
^33^	*	*	*	*	**	-	*	*	8/9
^35^	*	*	-	*	**	-	*	*	7/9
^37^	*	-	-	*	**	-	*	*	6/9
^38^	*	-	-	*	*	*	*	*	6/9

**Table 2:** Quality analysis based on the Jadad tool.

Author	1a- Randomized	1b- Concealment approp.	1c- Inappropriate	2a- Double Blind	2b- Appropriate	2c- Inappropriate	3- Follow-up	Total score
^31^	1	1	0	1	1	0	1	5/5
^34^	0	0	0	0	0	0	0	0/5
^36^	1	0	0	0	0	0	1	2/5

## DISCUSSION

The analyzed studies provided a comprehensive understanding of neuroplasticity in patients with ASD, investigating neurobiological markers and clinical correlations. The analysis of plasma proteins, such as α-synuclein and β-synuclein, revealed significant alterations in patients with ASD compared to the control group, suggesting a connection between underlying neurobiology and manifested clinical symptoms^33^. Genetic studies identified miRNAs and mutations in the SHANK2 gene as relevant factors in synaptic plasticity and neuronal development in patients with ASD, highlighting the complexity of the mechanisms related to this condition^38,39^. These findings are crucial for healthcare professionals and policymakers, offering essential information on the biological processes related to ASD and providing a solid foundation for the development of more targeted and effective therapeutic interventions.

The clinical correlations observed in the studies are fundamental for understanding the relationship between neurobiological characteristics and behavioral symptoms of ASD. For example, the reduction in face repetition suppression in autistic individuals correlated with the severity of social symptoms, emphasizing the interconnection between neurological expression and behavioral manifestations of ASD^42^.

Additionally, the relevance of BDNF as an indicator in understanding the pathophysiology of ASD development and progression contributes to advancing knowledge^43^. This finding complements subsequent studies that observed an elevation of this factor^35,44^.

These studies highlight the complexity of the mechanisms underlying ASD, suggesting an interconnection between hematological and neurobiological biomarkers, essential for developing targeted therapies^35,43,44^. BDNF has been studied in relation to autism, as it plays a crucial role in neuronal development and plasticity. It is suggested that BDNF levels may be altered in people with ASD^33,45^, which could influence various aspects of the condition, such as cognitive and behavioral development. However, this relationship is not yet fully understood, and more research is needed for better elucidation.

Moreover, the relevance of α-synuclein is highlighted as a fundamental tool for a better understanding of ASD pathophysiology and diagnosis, as this biochemical marker is differentiated in this population. Through these studies, a decrease in α-synuclein levels was observed in autistic individuals compared to healthy controls. Analyzing this potential biomarker could contribute to the early diagnosis of autism, in conjunction with clinical diagnosis, although it is not yet fully established.

These findings underscore the importance of addressing the neurobiological aspects of ASD to guide more precise intervention and diagnostic strategies, but it is important to note some limitations of this study. The analysis of metabolites induced by oxytocin administration was performed using peripheral blood samples, which may not fully reflect changes occurring in the central nervous system. Additionally, the absence of NCAM1 gene detection in children with ASD suggests the need for a larger sample to obtain more accurate data, especially during the critical period of neurodevelopment^34^. Despite the relevance of these biomarkers in the pathophysiology of ASD, it is important to highlight that the positive correlations suggest a potential relationship among them. However, due to the study's limitations, categorical statements cannot be made based on the results obtained.

In conclusion, the results of this research highlight the complexity of the neurobiological aspects underlying ASD by identifying distinct patterns of protein expression, genetic and anatomical variations, as well as relevant clinical correlations. The genetic analysis revealed an intricate interaction between miRNAs and genes associated with neuronal development and synaptic plasticity, while anatomical investigations indicated notable delays and specific characteristics in the brain development trajectories of individuals with ASD. Additionally, clinical correlations highlighted associations between visual hypersensitivity, gross motor skills, and symptom severity. This suggests that therapeutic approaches focused on neuroplasticity have the potential to modulate the neural circuits affected by ASD, thus providing promising perspectives for more effective and targeted therapeutic interventions. However, more specific studies are needed to evaluate the efficacy of these interventions.
