# Supplementary material for: Neuroplasticity in autism spectrum disorder: a systematic review
Source: Dement Neuropsychol. 2025 Jun 2;19:e20240182. doi: 10.1590/1980-5764-DN-2024-0182 (PMC12136586; doi:10.1590/1980-5764-DN-2024-0182)
Supplement: Supplementary file 1 [file 1980-5764-DN-19-e20240182-Suppl01.docx]

**Supplementary Material**

**Table S1.** Individual characterization of the studies.

| **Authors, year** | **Study design and location** | **Sample** | **Main results** | **Quality analysis** |
| --- | --- | --- | --- | --- |
| Sriwimol *et al.*, 2018[^34^](https://paperpile.com/c/JF2Ea7/qQom) | Case-control (Thailand) | 39 children with ASD and 29 children without ASD between 2 and 8 years old | The mean plasma α-synuclein level was significantly lower (P < 0.001) in children with ASD (10.82 ± 6.46 ng/mL) than in controls (29.47 ± 18.62 ng/mL), while the mean plasma β-synuclein level in children with ASD (1,344.19 ± 160.26 ng/mL) was significantly higher (P < 0.05) than in controls (1,219.16 ± 177.10 ng/mL). | High methodological quality |
| Jyonouchi *et al*., 2019[^39^](https://paperpile.com/c/JF2Ea7/qQom) | Case-control (United States) | 105 individuals with ASD aged between 2 and 21 years and 35 individuals without ASD aged between 4 and 30 years of both sexes. | 27 miRNAs were analyzed based on an IL-1ß/IL-10 ratio in ASD and non-ASD subgroups. It was observed that the genes targeted by miRNAs are enriched in specific signaling pathways such as neuronal development and synaptic plasticity (neurotrophin signaling and axon guidance pathways) in all ASD subgroups. | High methodological quality |
| Yang *et al.*, 2019[^35^](https://paperpile.com/c/JF2Ea7/qQom) | Case-control (China) | 40 children with ASD and 39 children without ASD between 2 and 6 years old | For the control group, the mean plasma NCAM1 level was 246.03 pg/mL, while in the children with ASD, the mean was 153.87 (p = 0.035), which was lower in the ASD group. As for neuropsychological development, NCAM1 levels were found to be positively correlated with gross motor ability (p = 0.41) and developmental quotient (p = 0.030) in children with ASD. | Moderate methodological quality |
| Zaslavsky *et al.*, 2019[^40^](https://paperpile.com/c/JF2Ea7/qQom) | Experimental (Canada) | Production of cortical neurons from pluripotent stem cells derived from donors without and with ASD | It has been observed that, in ASD, a reduced or mutated dosage of the SHANK2 gene causes an increase in synaptic connectivity, causing an increase in the length and complexity of the dendrite, the number of synapses and the frequency of spontaneous excitatory postsynaptic currents. | Low methodological quality |
| Bozkurt *et al.*, 2021[^36^](https://paperpile.com/c/JF2Ea7/qQom) | Transversal (Turkey) | 33 male children aged between 2 and 15 with ASD and 27 children without ASD with the same characteristics | The mean BDNF levels for the study group and the control group were 5.9 ± 2.8 ng/ml and 3.7 ± 1.8 ng/ml, respectively. Mean tPA levels for the study group and the control group were 32.9 ± 18.5 ng/ml and 25.5 ± 15.1 ng/ml, respectively. Cortisol, BDNF and tPA levels were significantly higher in the study group compared to the control group (p < 0.001). There was no statistically significant effect in terms of age, ABC total score and subscale on serum cortisol, BDNF and tPA levels in the study group (p > 0.05). | High methodological quality |
| Cai *et al.*, 2021[^43^](https://paperpile.com/c/JF2Ea7/qQom) | Transversal (China) | 441 ASD patients aged between 7 and 45 years and 426 age-matched healthy controls (HCs) using structural magnetic resonance neuroimaging from the ABIDE database. | The ASD patients exhibited a 7-year delay in reaching the maximum value compared to the control group in the cross-sectional trajectories of the network characteristics exhibited inverted U shapes; the ASD participants and the control group exhibited normalized mean degree differences in the right amygdala, and significant differences in the network characteristics were observed in the 18-year-old age group in most densities. | Moderate methodological quality |
| Ellis *et al*., 2021[^41^](https://paperpile.com/c/JF2Ea7/qQom) | Experimental (UK) | 16 individuals with ASD, aged between 19 and 67, and 15 individuals without ASD, aged between 20 and 66, of both sexes. | In the occipital region, individuals without ASD showed a significant improvement (p=0.034) in sensory cortical plasticity in the evoked potentials in the first and second test periods, while participants with ASD showed this effect only in the first test period. In the second period, significant differences were observed, with the group without ASD obtaining greater potentiation of the N1b component (p = 0.003), being similar in the parieto-occipital region in both groups (p = 0.010). In individuals with ASD, there was a significant association (p = 0.044) between visual hypersensitivity and the degree of potentiation in the first test period. | High methodological quality |
| Kato *et al.*, 2021[^37^](https://paperpile.com/c/JF2Ea7/qQom) | Multicenter randomized clinical trial (Japan)  Intervention: Intranasal administration of oxytocin (48 IU/day, 6 weeks) | 106 patients with ASD, aged between 18 and 54, 94 of whom were taking oxytocin alone and 12 were taking psychotropic drugs during the trial. | Among the 35 metabolites measured, a significant increase in N , N -dimethylglycine was detected in subjects given oxytocin compared to those given placebo with a medium effect size (corrected false discovery rate (FDR) P = 0.043, d = 0.74, N = 83). The increase in N , N -dimethylglycine was significantly correlated with oxytocin-induced clinical changes, assessed as changes in quantifiable features of autistic facial expression, including improvements between baseline and 2 weeks (P FDR = 0.006, r = - 0.485, N = 43) and deteriorations between 2 and 4 weeks (P FDR = 0.032, r = 0.415, N = 37). The results demonstrate an association of N , N -dimethylglycine up-regulation with the change over time in oxytocin efficacy in autistic social deficits and reinforce the involvement of the N -methyl-D-aspartate receptor and neural plasticity in the change over time in oxytocin efficacy. | High methodological quality |
| Desarkar *et al.*, 2022[^42^](https://paperpile.com/c/JF2Ea7/qQom) | Randomized crossover study (Canada)  Intervention: Repetitive Transcranial Magnetic Stimulation (rTMS; single session) | 31 autistic adults and 30 controls matched for sex, intelligence quotient and age. Autistic adults (n = 29) were randomized (1:1) with ages ranging from 18 to 50 years. | Both long-term potentiation (LTP) and long-term depression (LTD) increased significantly in the ASD group, indicating hyperplasticity. Active rTMS, but not simulated, attenuated LTD in autistic adults. | Low methodological quality |
| Al-Saei *et al.*, 2023[^38^](https://paperpile.com/c/JF2Ea7/qQom) | Clinical trial (Qatar and Spain) | 311 children with ASD, 247 boys and 64 girls; and 167 children with typical development, 94 boys and 73 girls | For subjects aged 5 to 12 years, the diagnostic algorithm with features, advanced glycation end products (AGEs) - N ε -carboxymethyl-lysine (CML), N ω -carboxymethylarginine (CMA) and hydroimidazolone derived from 3-deoxyglucosone (3DG-H) and oxidative damage marker, o '-dityrosine (DT), age and sex had an accuracy of 83% (CI 79 - 89%), sensitivity of 94% (CI 90-98%), specificity of 67% (CI 57-76%) and area under the curve of the receiver operating characteristic (AUROC) plot 0.87 (CI 0.84-0.90). The inclusion of additional adult plasma protein glycation and oxidation increased specificity to 74%. An algorithm with 12 plasma protein glycation and oxidation adult features was optimal for children aged 1.5 to 12 years: accuracy 74% (CI 70 to 79%), sensitivity 75% (CI 63 to 87%), specificity 74% (CI 58.) -90%) and AUROC 0.79 (CI 0.74-0.84). | High methodological quality |

Abbreviations: ASD, Autism spectrum disorder; NCAM1, Neuronal cell adhesion molecule-1; IL, Interleukin; BDNF, Brain-derived neurotrophic factor; tPA, Tissue plasminogen activator; miRNA, Micro Ribonucleic Acid; FDR, False discovery rate; LTP, Long-term potentiation; LTD, Long-term depression; rTMS, Repetition Transcranial Magnetic Stimulation; AGEs, advanced glycation end products; CML, N -ε -carboxymethyl-lysine; CMA, N ω -carboxymethylarginine; 3DG-H, hydroimidazolone derived from 3-deoxyglucosone; DT, oxidative damage marker, o , o '-dityrosine; AUROC, area under the curve of the receiver operating characteristic graph.

Source: Elaborated by the authors (2024).
